# miR-653-5p suppresses the viability and migration of fibroblast-like synoviocytes by targeting FGF2 and inactivation of the Wnt/beta-catenin pathway

**DOI:** 10.1186/s13018-021-02887-4

**Published:** 2022-01-04

**Authors:** Peilong Dong, Xiaobo Tang, Jian Wang, Botao Zhu, Zhiyun Li

**Affiliations:** Department of Orthopedics, Affiliated Jianhu Hospital of Nantong University, No. 666 Nanhuan Road, Jianhu, Yancheng, 224700 Jiangsu People’s Republic of China

**Keywords:** miR-653-5p, FGF2, Rheumatoid arthritis, Fibroblast-like synoviocytes

## Abstract

**Background:**

Rheumatoid arthritis (RA) is a chronic systemic autoimmune disease. Several studies reported that fibroblast-like synoviocytes (FLSs) and miRNAs are associated with RA pathogenesis. This study explored the function of miR-653-5p in the regulation of human fibroblast-like synoviocytes-rheumatoid arthritis (HFLS-RA) cells.

**Methods:**

The mRNA and protein levels of genes were measured by RT-qPCR and western blot, respectively. MTT, wound healing, and invasion assays were used to evaluate the viability and metastasis of FLSs. Luciferase reporter and RNA pull-down assays were employed to determine the interaction between miR-653-5p and FGF2.

**Results:**

RT-qPCR results demonstrated that miR-653-5p expression was decreased and FGF2 level was increased in synovial tissues and FLSs of RA. Moreover, the viability and metastasis of FLSs were accelerated by miR-653-5p addition, which was restrained by miR-653-5p suppression. Furthermore, we demonstrated that levels of Rac1, Cdc42, and RhoA were decreased after miR-653-5p addition. Besides, luciferase reporter and RNA pull-down assays implied that miR-653-5p targeted the 3′-UTR of FGF2. Functional assays showed that FGF2 overexpression neutralized the suppressive effects of miR-653-5p addition on HFLS-RA cell viability, metastasis, and the levels of Rho family proteins. Meanwhile, the levels of β-catenin, cyclin D1, and c-myc were declined by miR-653-5p supplementation, but enhanced by FGF2 addition.

**Conclusion:**

In sum, we manifested that miR-653-5p restrained HFLS-RA cell viability and metastasis via targeting FGF2 and repressing the Wnt/beta-Catenin pathway.

## Introduction

Rheumatoid arthritis (RA) is a heterogeneous and systemic autoimmune disease characterized by synovial cell inflammation and subsequent damage primarily to joint structures [1]. Fibroblast-like synovial cells (FLSs) are a key component in RA development [2, 3]. HFLS-RA cells present a series of invasive features, including enhanced proliferation, increased aggressiveness, and production of inflammatory mediators [4]. Previous studies reported that the lifetime risk of RA is 3.6% for women and 1.7% for men [5]. The current main clinical treatment strategy of RA is drug therapy, including immunosuppressive drugs and biologics. However, resistance to these therapies increases the risk of infectious diseases and cancer [6, 7]. Thus, it is necessary to look for new strategies for RA treatment.

MiRNAs are small non-coding RNA molecules (~ 20 nt) that bind to 3'-UTR of mRNA and modulate gene level at the post-transcriptional level, which have essential functions in musculoskeletal conditions, such as osteoarthritis and tendon injuries [8–11]. Moreover, increasing reports demonstrated that miRNAs play crucial roles in RA development [12]. For instance, miR-140-3p restrained the cell viability and induced apoptosis of synovial fibroblasts in RA via modulating sirtuin 3 [13]. Knockdown of PVT1 repressed the viability and inflammation of HFLS-RA cells through targeting miR-145-5p [14]. In a recent study, it was shown that upregulated miR-653-5p impeded the viability and promoted the apoptosis and inflammatory response of CHON-001 cells in osteoarthritis [15]. However, the role of miR-653-5p in RA progression is unclear.

Fibroblast growth factor 2 (FGF2), a member of the fibroblast growth factor (FGF) family, is implicated in multiple biological processes, such as cell proliferation, differentiation, and cell growth [16, 17]. A recent study reported that FGF2 was upregulated in chondrogenic differentiation and miR-23c repressed marrow stromal cell differentiation to chondrocytes through modulating FGF2 expression [17]. miR-105/Runx2 axis mediates FGF2-induced ADAMTS expression in osteoarthritis cartilage [18]. miR-16 modulated MgCl_2_-induced acceleration of osteogenic differentiation through regulating FGF2-mediated ERK/MAPK pathway activation [19]. Nonetheless, the mechanism of FGF2 in RA development remains elusive.

This study exhibited aberrant levels of miR-653-5p and FGF2 in RA patients and investigated the influence of miR-653-5p on RA development.

## Materials and methods

### Samples

Thirty-two synovial tissues were collected from RA patients (15 males, 17 females, 43–74 years old) after knee replacement surgeries at Affiliated Jianhu Hospital of Nantong University. Normal synovial biopsies from 32 patients with traumatic knee injuries served as healthy controls (18 males, 14 females, 42–72 years old). RA was diagnosed according to the previous reference standards [20]. The study was permitted by the Ethics Committee of Affiliated Jianhu Hospital of Nantong University, and written consent was gained from all patients.

### Cell culture and transfection

HFLS and HFLS-RA cells were purchased from Jennio Biotech Co., Ltd. (Guangzhou, China) and were cultured in DMEM containing 10% FBS (GBICO), 100 U/mL penicillin, and 100 μg/mL streptomycin in an incubator with 5% CO_2_ at 37 °C. miR-653-5p mimics/inhibitor and their negative controls (NC mimics/inhibitor), shFGF2, shNC, pcDNA3.1/FGF2, and pcDNA3.1 were generated by GenePhama (Shanghai, China). The plasmid vectors were transfected using Lipofectamine 2000 (Thermo Fisher Scientific).

### RT-qPCR

Total RNA was extracted from synovial tissues and HFLS-RA cells using Trizol reagent kits (Invitrogen). Then, 1 μg of total RNA was reverse transcribed to cDNA using the Revert Aid™ First Strand cDNA Synthesis kit (Takara) at 37˚C for 15 min. RT-qPCR was performed by SYBR Premix Ex Taq II (TaKaRa) on ABI 7500 real-time PCR system (Applied Biosystems). GAPDH (for mRNA) and U6 (for miR-653-5p) were used as an internal reference. Gene level was quantified by 2^−ΔΔCT^ method.

### MTT assay

MTT was conducted to assess HFLS-RA cell viability. In brief, 200 μl HFLS-RA cells (6 × 10^3^ cells/well) were seeded into 96-well plates and incubated with 10 μl MTT solution (Sigma) for 4 h at 37 °C. Then, the medium was removed and 150 μl DMSO was added to each well. Finally, the absorbance was determined at 490 nm using a spectrophotometric plate reader.

### Wound healing assay

Cell migration ability was performed using wound healing assay. HFLS-RA cells were seeded in 6-well plates and grown to full confluence. A 200 μl pipette tip was applied to generate artificial scratches. The wounded areas were observed and imaged by a microscope (Nikon, Japan).

### Transwell assays

HFLS-RA cell invasion was assessed using 8-μm-pore transwell chambers (BD Biosciences). HFLS-RA cells were seeded on the Matrigel chambers pre-coated with Matrigel. The lower chambers were filled with DMEM medium, and the upper chambers were filled with serum-free DMEM. After incubation for 24 h, cells were invaded to the lower chambers and stained with 0.1% crystal violet. Then, the cells were counted with a microscope (Olympus Corporation).

### Luciferase reporter assay

FGF2-(wild-type) wt and its mutant (FGF2-mut) were inserted into the pGL3 luciferase reporter vector (Promega,). Then, the above reporters were co-transfected miR-653-5p mimics or NC mimics into HFLS-RA cells for 48 h. The activities were measured by dual-luciferase reporter assay system (Promega).

### RNA pull-down

RNA pull-down was performed using a Magnetic RNA Pull-Down Kit (Thermo Fisher Scientific). The biotinylated miR-653-5p (Bio-miR-653-5p) and Bio-miR-NC were generated by RiboBio (Guangzhou, China). Then HFLS-RA cells were lysed and the protein lysates were incubated with M-280 streptavidin-coated with magnetic beads (Sigma-Aldrich) and then washed with buffer. Finally, RT-qPCRs were performed to determine gene expression.

### Western blot assay

Cells were lysed in RIPA buffer to isolate total proteins, and BCA protein kit (Sigma-Aldrich) measured the protein concentration. Protein lysates were separated by 10% SDS–PAGE and transferred to PVDF membranes (Bio-Rad, USA). After blocked with 5% skimmed milk, the membrane was incubated with primary antibodies against Rac1, Cdc42, RhoA, and β-catenin, cyclin D1, c-myc, or GAPDH, and then interacted with HRP-conjugated secondary antibodies. The protein bands were visualized with an ECL detection system (Millipore, USA).

### Statistical analysis

The data were exhibited as mean ± SD. Statistical analysis was conducted using SPSS 16.0 software (SPSS, IL, USA.). The student’s *t* test was applied for comparisons between two groups. One-way analysis of variance was carried out for comparisons among multiple groups. *P* < 0.05 indicated statistically significant.

## Results

### miR-653-5p level is decreased in synovial tissue and RA‑FLSs

**Fig. 1:**
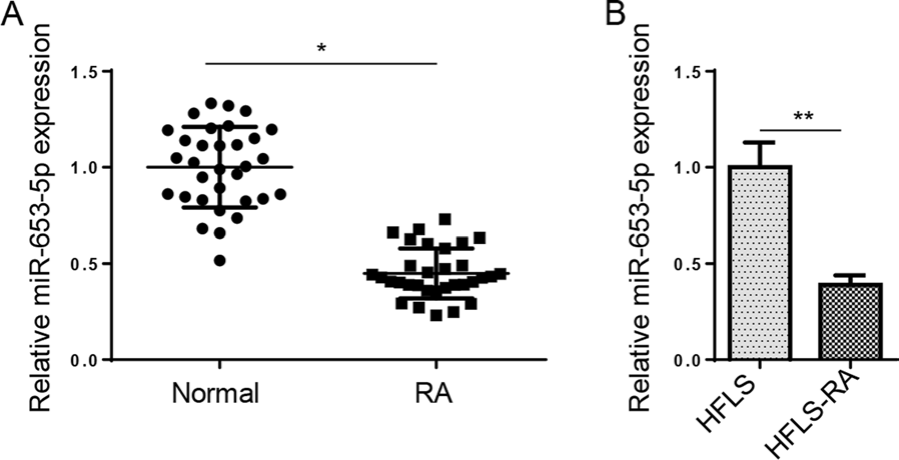
miR-653-5p level is decreased in synovial tissue and RA‑FLSs. **A** RT-qPCR was used to determine the expression levels of miR-653-5p in the synovial tissue of RA patients versus that of healthy synovial tissue. **B** RT-qPCR analysis showed the expression of miR-653-5p in HFLS-RA cells compared to that of healthy FLSs. *P < 0.05, **P < 0.01

Initially, RT-qPCR assay was adopted to determine miR-653-5p level in synovial tissues. We uncovered that miR-653-5p level was reduced in synovial tissues of RA patients (Fig. 1A). Consistently, we also identified that miR-653-5p level was decreased in HFLS-RA cells compared with the normal HFLS cells (Fig. 1B). Hence, these results manifested that miR-653-5p might act as a vital role in RA pathogenesis.

### The addition of miR-653-5p restrains the viability, migration, and invasion in HFLS-RAs

**Fig. 2:**
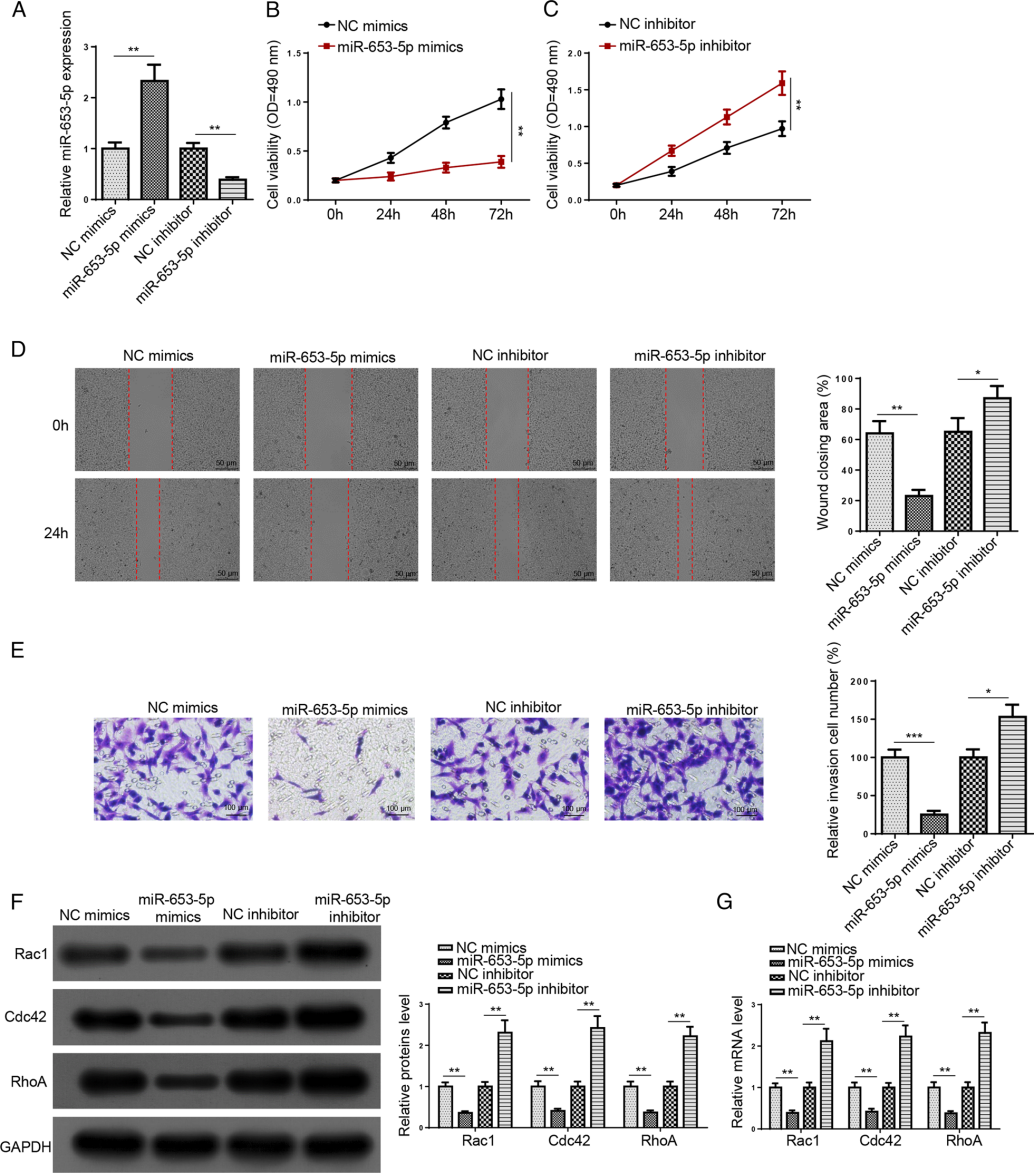
The addition of miR-653-5p restrains the viability, migration, and invasion in HFLS-RA cells. **A** RT-qPCR analysis showed the expression of miR-653-5p in HFLS-RA cells transfection of miR-653-5p mimics or NC mimics and miR-653-5p inhibitor or NC inhibitor. **B** and **C** MTT assay showed cell viability in HFLS-RA cells transfection of miR-653-5p mimics or NC mimics and miR-653-5p inhibitor or NC inhibitor. **D** and **E** HFLS-RA cell migration and invasion were measured using wound healing and transwell assays after transfection with miR-653-5p mimics or NC mimics and miR-653-5p inhibitor or NC inhibitor. **F** and **G** The protein and mRNA expression of Rac1, Cdc42, and RhoA in HFLS-RA cells transfection of miR-653-5p mimics or NC mimics and miR-653-5p inhibitor or NC inhibitor was detected by western blot and RT-qPCR assay. *P < 0.05, **P < 0.01, ***P < 0.001

Subsequently, we explored the function of miR-653-5p in RA. RT-qPCR results elaborated the miR-653-5p level was heightened by miR-653-5p addition and was reduced by miR-653-5p silencing (Fig. 2A). Moreover, cell viability was inhibited in HFLS-RA cells transfected with miR-653-5p mimics, whereas miR-653-5p deletion reversed this effect (Fig. 2B, C). Meanwhile, we found that miR-653-5p addition restrained the migration and invasion of HFLS-RA cells; however, miR-653-5p knockdown exhibited a contrary effect (Fig. 2D, E). Also, we uncovered that miR-653-5p mimics decreased Rac1, Cdc42, and RhoA levels, which was increased by miR-653-5p deletion (Fig. 2F, G). Above all, these data suggested that miR-653-5p restrained HFLS-RA cell viability, migration, and invasion.

### FGF2 is the direct target of miR-653-5p

**Fig. 3:**
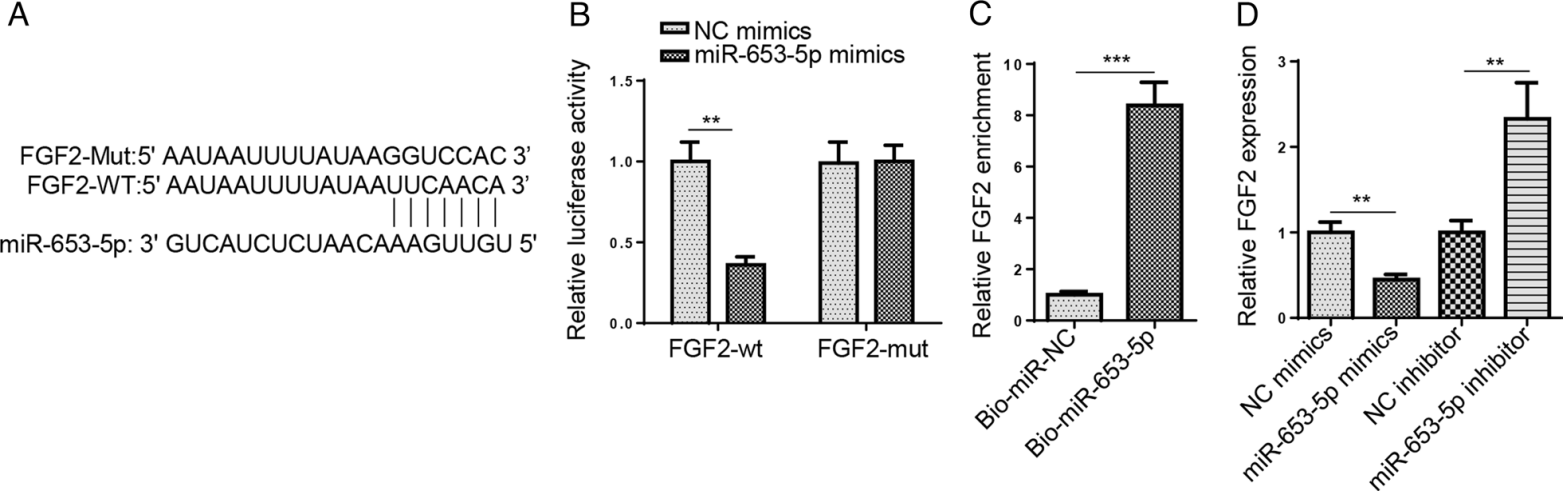
FGF2 is the direct target of miR-653-5p. **A** The complementary sequences of miR-653-5p and corresponding sequence of the 3'‑UTR of FGF2 was predicted by starBase website. **B** Luciferase reporter assay showed luciferase activity of FGF2-WT or FGF2-MUT in HFLS-RA cells transfected with NC mimics or miR-653-5p mimics. **C** Association between miR-653-5p and FGF2 was determined using an RNA pull-down assay. **D** RT‑qPCR analysis was used to evaluate the expression of FGF2 in HFLS-RA cells transfection of miR-653-5p mimics or NC mimics and miR-653-5p inhibitor or NC inhibitor. **P < 0.01, ***P < 0.001

We then predicted miR-653-5p target genes with starBase and uncovered that FGF2 was a potential target of miR-653-5p. The binding sites are shown in Fig. 3A. Then, luciferase reporter assay elaborated that miR-653-5p mimics repressed the luciferase activity of FGF2-wt, but had no effect on FGF2-mut (Fig. 3B). Moreover, pull-down assay implied that FGF2 could bind with miR-653-5p (Fig. 3C). Furthermore, the FGF2 level was decreased by miR-653-5p supplementation, which was heightened by miR-653-5p inhibition (Fig. 3D). Collectively, we determined that FGF2 was a target of miR-653-5p.

### FGF2 deletion suppresses RA development

**Fig. 4:**
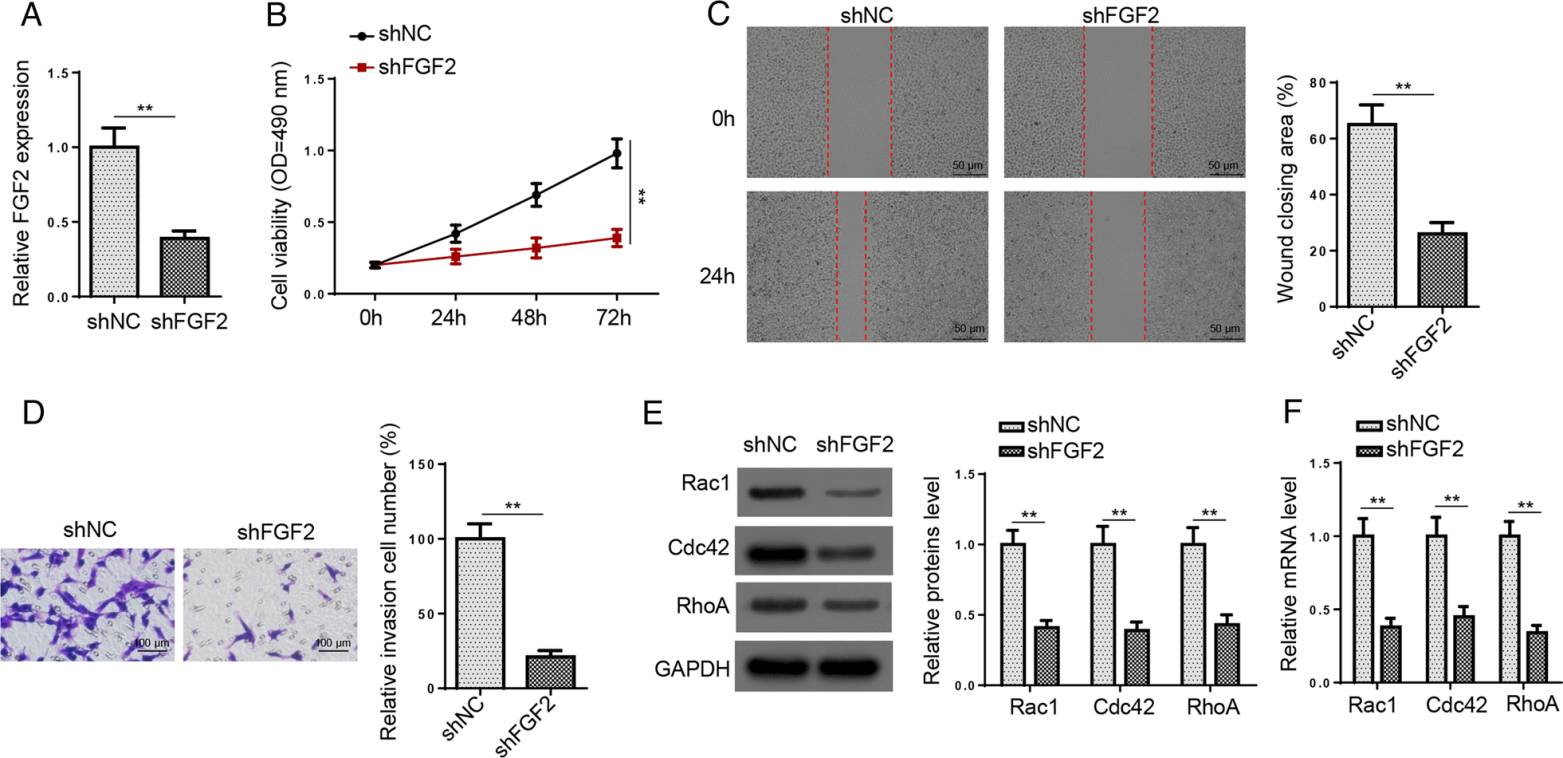
FGF2 deletion suppresses RA development. **A** RT-qPCR was used to determine FGF2 expression in HFLS-RA cells transfected with shFGF2 or shNC. **B**–**D** MTT, wound healing, and transwell assays showed cell viability, migration, and invasion in HFLS-RA cells transfection of shFGF2 or shNC. **E** and **F** Western blot and RT-qPCR assays showed the protein and mRNA expression of Rac1, Cdc42, and RhoA in HFLS-RA cells transfected with shFGF2 or shNC. **P < 0.01

To further verify the function of FGF2 in RA, HFLS-RA cells were transfected with shFGF2. Results elaborated that FGF2 was downregulated by FGF2 deficiency (Fig. 4A). Meanwhile, cell viability, migration, and invasion were suppressed after FGF2 depletion (Fig. 4B–D). Similarly, we uncovered that the mRNA and protein levels of Rac1, Cdc42, and RhoA were both decreased by FGF2 silence (Fig. 4E, F). As a result, FGF2 deletion diminished HFLS-RA cell viability and metastasis.

### miR-653-5p regulates HFLS-RA cell viability and metastasis via targeting FGF2

**Fig. 5:**
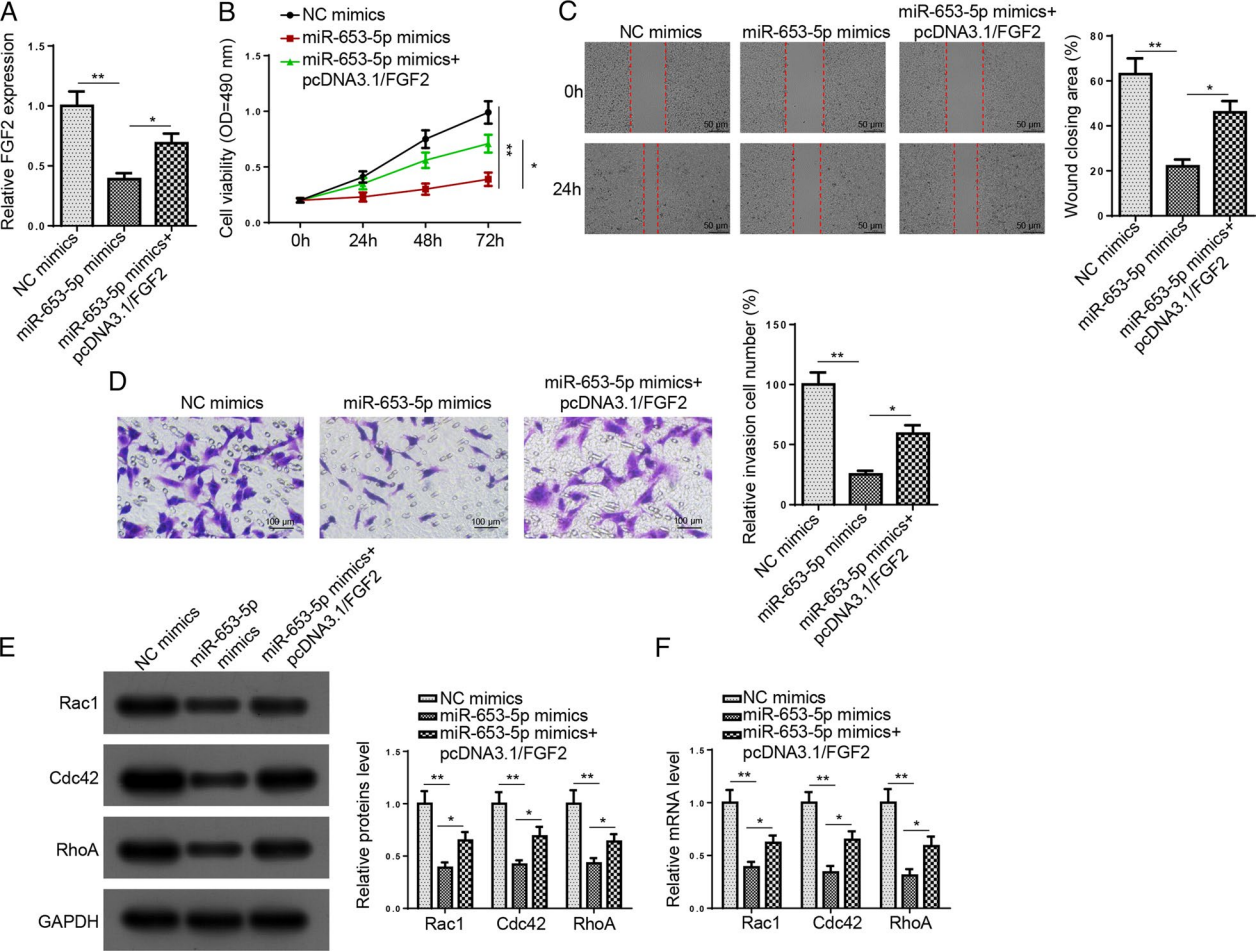
miR-653-5p regulates HFLS-RA cell viability and metastasis via targeting FGF2. **A** RT-qPCR determined FGF2 expression in HFLS-RA cells transfected with NC mimics, miR-653-5p mimics, miR-653-5p mimics + pcDNA3.1/FGF2. **B**–**D** MTT, wound healing, and transwell assays showed cell viability, migration, and invasion in HFLS-RA cells transfection of NC mimics, miR-653-5p mimics, miR-653-5p mimics + pcDNA3.1/FGF2. **E** and **F** Western blot and RT-qPCR assays showed the protein and mRNA expression of Rac1, Cdc42, and RhoA in HFLS-RA cells transfected with NC mimics, miR-653-5p mimics, miR-653-5p mimics + pcDNA3.1/FGF2. *P < 0.05, **P < 0.01

To further determine whether miR-653-5p exerted its function via FGF2, HFLS-RA cells were transfected with NC mimics, miR-653-5p mimics, miR-653-5p mimics + pcDNA3.1/FGF2. Figure 5A displays that the FGF2 level was inhibited by miR-653-5p addition, while FGF2 overexpression reversed this effect. Functional assays revealed that upregulated FGF2 rescued the repression effects of miR-653-5p supplementation on HFLS-RA cell viability, migration, and invasion (Fig. 5B–D). Moreover, FGF2 overexpression rescued the suppressive effect on the levels of Rac1, Cdc42, and RhoA caused by miR-653-5p addition (Fig. 5E, F). In sum, miR-653-5p modulated RA progression via targeting FGF2.

### miR-653-5p/FGF2 restrains the Wnt/β-catenin pathway in HFLS-RA cells

**Fig. 6:**
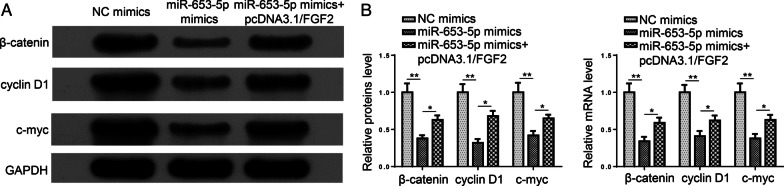
miR-653-5p/FGF2 restrains the Wnt/β-Catenin pathway in HFLS-RA cells. **A** and **B** RT-qPCR and western blot analysis showed the levels of β-catenin, cyclin D1 and c-myc in HFLS-RA cells transfected with NC mimics, miR-653-5p mimics, miR-653-5p mimics + pcDNA3.1/FGF2. *P < 0.05, **P < 0.01

Wnt/β-catenin signaling can be mediators in RA biological processes [21, 22]. Previous studies reported that FGF2 is a modulator of Wnt/β-catenin pathway. Thus, we speculated that miR-653-5p/FGF2 regulated RA progression via regulating the Wnt/β-catenin pathway. Results elaborated that β-catenin, cyclin D1, and c-myc levels were reduced by miR-653-5p addition, while FGF2 overexpression reversed these effects (Fig. 6A, B). These data manifested that miR-653-5p targeted FGF2 and inhibited the Wnt/β-catenin pathway in HFLS-RA cells.

## Conclusion

Accumulating evidence has reported that miRNAs are closely related to RA occurrence and development, such as miR-140-3p [23], miRNA-146a [24], and miR-17-5p [25]. Therefore, miRNAs were considered as a potential therapeutic target for RA. For example, it was reported that miR-6089 restrained HFLS-RA cell viability and promoted apoptosis through regulating CCR4 [26]. MiR-421 accelerated the inflammatory response of FLS s in RA via targeting SPRY1 [27]. This research explored the effect of miR-653-5p on the viability, migration, and invasion of HFLS-RA cells.

HFLS-RA cells are the main cell population involved in RA progression of synovial tissues. Previous researches exhibited that inhibiting FLS migration and invasion may protect RA joint destruction [28]. Thus, regulating the migration and invasion of HFLS-RAs may be a new strategy for RA treatment. Moreover, miR-653-5p was identified to impede cell migrative and invasive ability in breast cancer [29]. Our work suggested that miR-653-5p restrained cell migration and invasion in HFLS-RA cells.

Rho family proteins participated in regulating HFLS-RA cell viability and invasion. For instance, RhoA is considered a new target for modulating HFLS-RA cell invasion [30]. Rac1 activation facilitated proliferation and mediates IL-17A-induced HFLS-RA cell migration [31, 32]. This led us to propose that miR-653-5p played a vital role through Rho proteins in HFLS-RA cells. This study elucidated that miR-653-5p addition inhibited RhoA, Rac1, and Cdc42 protein levels in HFLS-RA cells.

Multiple miRNAs exerted their biological functions via modulating their target molecules. miR-653-5p was identified to target different genes, such as EMSY [33], and RAI14 [34], which participate in many tumor developments. In this work, we determined that FGF2 was the target of miR-653-5p in HFLS-RA cells. FGF2 is correlated with multiple biological processes including tumor growth, apoptosis, and angiogenesis [35, 36]. Importantly, FGF2 was found to be upregulated in RA patients and its level was closely related to Larsen’s grade of bone erosion [37, 38]. Herein, we revealed that FGF2 addition alleviated the repressive influence of miR-653-5p supplementation on the viability and metastasis of HFLS-RA cells as well as on Rho family protein levels. Moreover, β-catenin, cyclin D1, and c-myc levels were decreased by miR-653-5p addition, which was increased by FGF2 addition. Taken together, miR-653-5p/FGF2 axis inhibited the Wnt/β-catenin pathway of HFLS-RA cells.

## Conclusion

The current study illustrated that miR-653-5p restrained RA progression through targeting FGF2 and inactivation of the Wnt/β-catenin pathway, indicating that miR-653-5p may be an effective treatment target for RA.

## Data Availability

The datasets used and/or analyzed during the current study are available from the corresponding author on reasonable request.

## Abbreviations

RA: Rheumatoid arthritis
FLSs: Fibroblast-like synovial cells
HFLS-RA: Human fibroblast-like synoviocytes-rheumatoid arthritis
FGF2: Fibroblast growth factor 2
Rac1: Rac family small GTPase 1
Cdc42: Cell division cycle 42
RhoA: Ras homolog family member A

## References

[1] Mateen S, Zafar A, Moin S, Khan AQ, Zubair S (2016). Understanding the role of cytokines in the pathogenesis of rheumatoid arthritis. Clin Chim Acta.

[2] Rockel JS, Kapoor M (2017). Autophagy: controlling cell fate in rheumatic diseases. Nat Rev Rheumatol.

[3] Bartok B, Hammaker D, Firestein GS (2014). Phosphoinositide 3-kinase delta regulates migration and invasion of synoviocytes in rheumatoid arthritis. J Immunol.

[4] Turner JD, Filer A (2015). The role of the synovial fibroblast in rheumatoid arthritis pathogenesis. Curr Opin Rheumatol.

[5] Crowson CS, Matteson EL, Myasoedova E, Michet CJ, Ernste FC, Warrington KJ, Davis JM, Hunder GG, Therneau TM, Gabriel SE (2011). The lifetime risk of adult-onset rheumatoid arthritis and other inflammatory autoimmune rheumatic diseases. Arthritis Rheum.

[6] Flores-Borja F, Mauri C, Ehrenstein MR (2008). Restoring the balance: harnessing regulatory T cells for therapy in rheumatoid arthritis. Eur J Immunol.

[7] Imperato AK, Bingham CO, Abramson SB (2004). Overview of benefit/risk of biological agents. Clin Exp Rheumatol.

[8] He L, Hannon GJ (2004). MicroRNAs: small RNAs with a big role in gene regulation. Nat Rev Genet.

[9] Giordano L, Porta GD, Peretti GM, Maffulli N (2020). Therapeutic potential of microRNA in tendon injuries. Br Med Bull.

[10] Oliviero A, Della Porta G, Peretti GM, Maffulli N (2019). MicroRNA in osteoarthritis: physiopathology, diagnosis and therapeutic challenge. Br Med Bull.

[11] Gargano G, Oliviero A, Oliva F, Maffulli N (2021). Small interfering RNAs in tendon homeostasis. Br Med Bull.

[12] Filkova M, Jungel A, Gay RE, Gay S (2012). MicroRNAs in rheumatoid arthritis: potential role in diagnosis and therapy. BioDrugs.

[13] Zu B, Liu L, Wang J, Li M, Yang J (2021). MiR-140-3p inhibits the cell viability and promotes apoptosis of synovial fibroblasts in rheumatoid arthritis through targeting sirtuin 3. J Orthop Surg Res.

[14] Tang J, Yi S, Liu Y (2020). Long non-coding RNA PVT1 can regulate the proliferation and inflammatory responses of rheumatoid arthritis fibroblast-like synoviocytes by targeting microRNA-145-5p. Hum Cell.

[15] Lian LP, Xi XY (2020). Long non-coding RNA XIST protects chondrocytes ATDC5 and CHON-001 from IL-1beta-induced injury via regulating miR-653-5p/SIRT1 axis. J Biol Regul Homeost Agents.

[16] Chen F, Qi S, Zhang X, Wu J, Yang X, Wang R (2019). miR-23a-3p suppresses cell proliferation in oral squamous cell carcinomas by targeting FGF2 and correlates with a better prognosis: miR-23a-3p inhibits OSCC growth by targeting FGF2. Pathol Res Pract.

[17] Shen PF, Wang B, Qu YX, Zheng C, Xu JD, Xie ZK, Ma Y (2019). MicroRNA-23c inhibits articular cartilage damage recovery by regulating MSCs differentiation to chondrocytes via reducing FGF2. Eur Rev Med Pharmacol Sci.

[18] Ji Q, Xu X, Xu Y, Fan Z, Kang L, Li L, Liang Y, Guo J, Hong T, Li Z (2016). miR-105/Runx2 axis mediates FGF2-induced ADAMTS expression in osteoarthritis cartilage. J Mol Med (Berl).

[19] Qi H, Liu Y, Wu L, Ni S, Sun J, Xue J, Liu Q, Ni X, Fan W (2020). MicroRNA-16, via FGF2 regulation of the ERK/MAPK pathway, is involved in the magnesium-promoted osteogenic differentiation of mesenchymal stem cells. Oxid Med Cell Longev.

[20] Aggarwal R, Rider LG, Ruperto N, Bayat N, Erman B, Feldman BM, Oddis CV, Amato AA, Chinoy H, Cooper RG (2017). 2016 American College of Rheumatology/European League Against Rheumatism criteria for minimal, moderate, and major clinical response in adult dermatomyositis and polymyositis: an International Myositis Assessment and Clinical Studies Group/Paediatric Rheumatology International Trials Organisation Collaborative Initiative. Ann Rheum Dis.

[21] Li GQ, Fang YX, Liu Y, Meng FR, Wu X, Zhang CW, Zhang Y, Liu D, Gao B (2019). MALAT1-driven inhibition of Wnt signal impedes proliferation and inflammation in fibroblast-like synoviocytes through CTNNB1 promoter methylation in rheumatoid arthritis. Hum Gene Ther.

[22] Wu J, Fan W, Ma L, Geng X (2018). miR-708-5p promotes fibroblast-like synoviocytes' cell apoptosis and ameliorates rheumatoid arthritis by the inhibition of Wnt3a/beta-catenin pathway. Drug Des Devel Ther.

[23] Wangzhou K, Lai Z, Lu Z, Fu W, Liu C, Liang Z, Tan Y, Li C, Hao C (2020). MiR-143–3p Inhibits osteogenic differentiation of human periodontal ligament cells by targeting KLF5 and inactivating the Wnt/beta-catenin pathway. Front Physiol.

[24] Tavasolian F, Hosseini AZ, Soudi S, Naderi M (2020). miRNA-146a improves immunomodulatory effects of MSC-derived exosomes in rheumatoid arthritis. Curr Gene Ther.

[25] Najm A, Masson FM, Preuss P, Georges S, Ory B, Quillard T, Sood S, Goodyear CS, Veale DJ, Fearon U (2020). MicroRNA-17-5p reduces inflammation and bone erosions in mice with collagen-induced arthritis and directly targets the JAK/STAT pathway in rheumatoid arthritis fibroblast-like synoviocytes. Arthritis Rheumatol.

[26] Lin S, Wang S, Zhang Z, Lu Y, Yang M, Chen P, Chen L, Wang M (2020). MiRNA-6089 inhibits rheumatoid arthritis fibroblast-like synoviocytes proliferation and induces apoptosis by targeting CCR4. Arch Physiol Biochem.

[27] Jiang F, Zhou HY, Zhou LF, Wen YH, Gai HH, Wu GM (2019). MicroRNA-421 promotes inflammatory response of fibroblast-like synoviocytes in rheumatoid arthritis by targeting SPRY1. Eur Rev Med Pharmacol Sci.

[28] Lao M, Shi M, Zou Y, Huang M, Ye Y, Qiu Q, Xiao Y, Zeng S, Liang L, Yang X (2016). Protein inhibitor of activated STAT3 regulates migration, invasion, and activation of fibroblast-like synoviocytes in rheumatoid arthritis. J Immunol.

[29] Zhang M, Wang H, Zhang X, Liu F (2021). miR6535p suppresses the growth and migration of breast cancer cells by targeting MAPK6. Mol Med Rep.

[30] Xiao Y, Liang L, Pan Y, Lian F, Li L, Lin H, Fu D, Fan J, Yang X, Sun L (2013). Inhibitory effects of simvastatin on migration and invasion of rheumatoid fibroblast-like synoviocytes by preventing geranylgeranylation of RhoA. Rheumatol Int.

[31] Chan A, Akhtar M, Brenner M, Zheng Y, Gulko PS, Symons M (2007). The GTPase Rac regulates the proliferation and invasion of fibroblast-like synoviocytes from rheumatoid arthritis patients. Mol Med.

[32] Kanai Y, Dohmae N, Hirokawa N (2004). Kinesin transports RNA: isolation and characterization of an RNA-transporting granule. Neuron.

[33] Yan A, Chen G, Nie J. DGUOK-AS1 promotes the proliferation cervical cancer through regulating miR-653-5p/EMSY. Cancer Biol Ther 2020:1–9. 10.1080/15384047.2020.1775445 PMC8726694 32660328

[34] Liu F, Hu L, Pei Y, Zheng K, Wang W, Li S, Qiu E, Shang G, Zhang J, Zhang X (2020). Long non-coding RNA AFAP1-AS1 accelerates the progression of melanoma by targeting miR-653-5p/RAI14 axis. BMC Cancer.

[35] Sun LL, Lei FR, Jiang XD, Du XL, Xiao L, Li WD, Li XQ (2020). LncRNA GUSBP5-AS promotes EPC migration and angiogenesis and deep vein thrombosis resolution by regulating FGF2 and MMP2/9 through the miR-223-3p/FOXO1/Akt pathway. Aging (Albany NY).

[36] Li R, Wang Y, Xu Y, He X, Li Y (2019). Silencing the long noncoding RNA, TINCR, a molecular sponge of miR335, inhibits the malignant phenotype of epithelial ovarian cancer via FGF2 suppression. Int J Oncol.

[37] Manabe N, Oda H, Nakamura K, Kuga Y, Uchida S, Kawaguchi H (1999). Involvement of fibroblast growth factor-2 in joint destruction of rheumatoid arthritis patients. Rheumatology (Oxford).

[38] Qu Z, Huang XN, Ahmadi P, Andresevic J, Planck SR, Hart CE, Rosenbaum JT (1995). Expression of basic fibroblast growth factor in synovial tissue from patients with rheumatoid arthritis and degenerative joint disease. Lab Invest.

